# Une éruption pustuleuse

**DOI:** 10.11604/pamj.2017.27.63.10807

**Published:** 2017-05-29

**Authors:** Hasnaa Zaouri, Baderdine Hassam

**Affiliations:** 1 Service de Dermatologie, CHU Ibn Sina, Rabat-Instituts, rue Famfdal Cherkaoui, BP 6527, 10000 Rabat, Maroc

**Keywords:** Pustulose amicrobienne, hypocalcémie, maladies à expression cutanée, Amicrobial pustulosis, hypocalcemia, skin diseases

## Image en médecine

Les pustuloses amicrobiennes (PA) correspondent à un groupe de maladies à expression cutanée dont la lésion élémentaire est une pustule. Elles sont actuellement considérées comme une maladie rare associée à un large spectre de troubles immunitaires. Cliniquement,ellessont caractérisées par des récurrences d’éruptions pustuleuses aseptiques, siégeant principalement au niveau du cuir chevelu, les grands plis et le tronc. L’histologie permetde différencier entre les pustules uniloculaires (non spongiforme) et les pustules multiloculaires (spongiformes). Les PA ont été décrite en association avec le lupus érythémateux systémique, la myasthénie, le syndrome de Sjögren, la maladie coeliaque, l’impétigo herpétiforme, le psoriasis ou une pustulose exanthématique aigue généralisée. Mais avant de réaliser un bilan étiologique, il faut éliminer une hypocalcémie. Toutes les causes d’hypocalcémie peuvent provoquer une PA: hypoparathyroïdie, malabsorption, insuffisance rénale chronique, dans ce cas, la correction de l'hypocalcémie entraîne la guérison.Nous rapportons le cas d’un patient âgé de 33 ans, suivi pour une néphropathie glomérulaire primitive stade d’insuffisance rénale chronique, qui présente depuis une semaine une éruption généralisée, faite de lésions pustuleuses à contenus blanchâtres, de taille variable, évoluant dans un contexte d’apyrexie (A). Le prélèvement bactériologique d’une pustule était stérile. L'examen histopathologique a montré des pustules spongiformes multiloculaires, avec infiltrat inflammatoire neutrophilique interstitiel. Le bilan biologique a révélé une hypocalcémie. La correction de l'hypocalcémie a permis la régression des lésions au bout d'une semaine (B).

**Figure 1:**
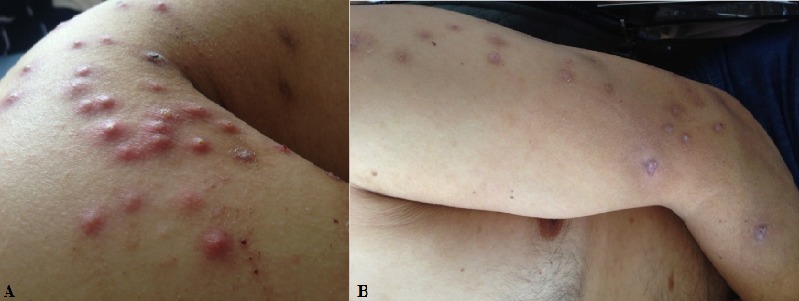
A) lésions pustuleuses de contenus blanchâtres; B) régression des lésions après correction de l’hypocalcémie

